# Right to attention to sexuality for people with mental disorders: bridges between health and social services

**DOI:** 10.1192/j.eurpsy.2024.1489

**Published:** 2024-08-27

**Authors:** M. T. Campillo Sanz, M. Vallve Elias, A. Casals Arnau, J. Marti Bonany, D. Garcia Hernandez, R. Sanchez Gonzalez

**Affiliations:** Salut Mental Institut, Hospital del Mar, Barcelona, Spain

## Abstract

**Introduction:**

The expression of sexuality in the adult with mental disorders depends on the early incorporation of factors for promoting social inclusion. It is fundamental that sexual educators and advisors, in addition to working with the clients, also work with close family members. Intervention programs should establish objectives for developing a positive attitude towards sexuality in people with mental disorders and improving self-esteem (Katz G,Salud Publica Mex. 2008;50 Suppl 2:s239-54).

**Challenge:**

Achieving support for people with mental health problems and/or substance use disorder admitted to the Social Rehabilitation Process of a psychiatric hospital so that they develop their sexuality satisfactorily. The right to privacy must be taken into account.

**Objectives:**

Promoting a healthy and satisfactory development of sexuality in people with severe mental disorders. Raising awareness among healthcare teams, families and legal representatives regarding the need and suitability for support. Introducing the concept of sexuality as a dignifying perspective. Promoting sexual education that avoids disadvantages and situations of abuse in the target group. Coordinating the continuity of the project with non-health social services after discharge.

**Hypothesis:**

Possibility of receiving support in the development of sexuality through training, information and improvement in the management of emotions/feelings in people who express the need or willingness to receive it, will contribute to overcoming limitations or difficulties.

**Methods:**

Detecting people who during 2021 wish to work on the objectives through the care team. Searching for community resources aimed at attending sexuality issues in people with mental health problems. Proposing the hospital a collaboration with a non-profit entity that develops a specialized program for attention to sexuality in disability. Coordination between Treatment team and Entity. Quantitative and qualitative assessment of one year of experience according to the parameters of the entity.

**Results:**

**Table:** 

2022	People included	Percentage of people admitted to the Income Unit (65)
Detection concern sex-affectivity	5	7,69%
Verbalized concern	3	4,61%
Referral to the entity program	2	3,07%

**Conclusions:**

All patients included have a diagnosis of psychosis. Experience was very positive for the participants. Community intervention projects that lead to an education in healthy and respectful relationships in the field of sexuality and affectivity are necessary. This would allow to prevent behaviours and situations at risk of abuse as well as social and emotional instability.

**Disclosure of Interest:**

None Declared

